# Testing the Magnitude of Correlations Across Experimental Conditions

**DOI:** 10.3389/fpsyg.2022.860213

**Published:** 2022-05-26

**Authors:** Simone Di Plinio

**Affiliations:** Department of Neuroscience Imaging and Clinical Sciences, “G. D’Annunzio” University of Chieti-Pescara, Chieti, Italy

**Keywords:** correlation, bootstrap, effect size, *p*-value, mixed-effects, sample size, fisher, Cohen

## Abstract

Correlation coefficients are often compared to investigate data across multiple research fields, as they allow investigators to determine different degrees of correlation to independent variables. Even with adequate sample size, such differences may be minor but still scientifically relevant. To date, although much effort has gone into developing methods for estimating differences across correlation coefficients, adequate tools for variable sample sizes and correlational strengths have yet to be tested. The present study evaluated four different methods for detecting the difference between two correlations and tested the adequacy of each method using simulations with multiple data structures. The methods tested were Cohen’s q, Fisher’s method, linear mixed-effects models (LMEM), and an *ad hoc* developed procedure that integrates bootstrap and effect size estimation. Correlation strengths and sample size was varied across a wide range of simulations to test the power of the methods to reject the null hypothesis (i.e., the two correlations are equal). Results showed that Fisher’s method and the LMEM failed to reject the null hypothesis even in the presence of relevant differences between correlations and that Cohen’s method was not sensitive to the data structure. Bootstrap followed by effect size estimation resulted in a fair, unbiased compromise for estimating quantitative differences between statistical associations and producing outputs that could be easily compared across studies. This unbiased method is easily implementable in MatLab through the bootes function, which was made available online by the author at MathWorks.

## Introduction

Comparing statistics is a frequent point of contention among researchers. The need to compare correlations is common and requires a specific assay to determine whether a continuous variable, often called covariate, has a different degree of correlation between two sets of data. Examples of fields in need of such an assay include cognitive psychology (e.g., the correlation between the degree of task automation and behavioral performance in extroverted vs. introverted individuals), social psychology (e.g., the correlation between social exclusion and job satisfaction in men vs. women), and cognitive neuroscience (e.g., the correlation between brain activity and behavioral performance under negative vs. positive emotional stimulation). Calculated differences may be minor, even with the recommended sample size, but can still be scientifically relevant (Ellis, 2010). Furthermore, modern science is gradually moving away from p-centric data interpretation toward effect-size-oriented approaches (Kelley and Preacher, 2012; Sullivan and Feinn, 2012). Thus, reporting only *r*- and *p*-values and binarizing result interpretations as either significant or non-significant, depending on an (eventually corrected) threshold of *p* < 0.05, has become outdated (Nichols et al., 2017; Ioannidis, 2019).

The existence of a statistical association cannot be relied upon to evaluate whether the strength of the relation between two variables will always be the same. In a within-group design, the correlation coefficients between the outcome (DV) and the covariate (IV) may be decreased in a specific experimental condition B than in another condition A. Alternatively, in a between-group design, the treatment group may show a weaker correlation between the outcome and the covariate than in the control group, or vice versa. These context-dependent or group-dependent effects on the extent of the correlation between two variables should be investigated using the most suited methods so that scientists from different disciplines can assess the most fitting comparison between the two correlations. Of note, given that null hypotheses are always false when evaluated with large datasets (Cohen, 1990), and that both small samples and large samples can convey useful information (Lindquist et al., 2012; Friston, 2013), the effect of a correlational change should be investigated not only with various correlation values, but also various sample sizes. However, even though much effort has been invested into developing methodologies that can estimate differences across correlation coefficients, studies investigating this problem using a comprehensive approach and variable sample sizes have yet to be published.

Several strategies have been developed to estimate differences between correlations. The simplest method was proposed by Cohen (1988) and estimated an effect size as the difference between two Fisher-transformed correlations. Fisher’s method (Fisher, 1921) also accounts for sample size and calculates the probability that two correlations will differ given their strength and the number of samples in the two groups. Whereas Cohen’s and Fisher’s methods rely exclusively on *r*-values, ignoring the initial data structure, analysis of covariance (ANCOVA) and linear mixed-effects models (LMEM) retain this information. ANCOVA and LMEMs have been widely adopted for analyzing data in cognitive neuroscience experiments, wherein the parameters observed are affected by multiple factors (Buckner et al., 2008; Garrett et al., 2010; Zilles and Amunts, 2013).

A bootstrap approach followed by calculation of the effect size may also be used to detect changes in correlations between neurophysiological parameters and behavioral performance across experimental conditions (Di Plinio et al., 2018). This method allowed testing the hypothesis that the association between functional connectivity across brain regions and behavioral performance (Hampson et al., 2010) was weakened by negative emotional stimulation. This approach (bootstrap and effect size estimation) is particularly advantageous since it is less impaired by violating normality assumptions (Liu and Popmey, 2020). Finally, structural equation modeling has also been proposed for testing independent or dependent correlational hypotheses (Cheung and Chan, 2004). This method is grounded in confirmatory factor analysis and is useful when data includes both dependent and independent measures. However, this approach may be ill-suited to small sample sizes and is more appropriate for meta-analytic designs (Cheung and Chan, 2005; Cheung, 2014).

This paper examines four different approaches to evaluate their power to detect an effect. The term effect in this study refers to “a change in the correlation between two conditions or two groups.” Although *p*-values are undergoing a theoretical revision by the scientific community, they still provide a universally recognized statistic (Goodman, 2019; Greenland, 2019). As such, both *p*-values and effect estimates are reported for each method. The methods examined in this study are Cohen’s q, Fisher’s method, LMEM, and bootstrap with effect size estimation. A series of simulations were implemented to test the four methods in an environment wherein correlational strength and sample size vary from one cycle to the next. Method performances are then discussed.

## Materials and Methods

### Simulation Parameters

In the simulations used in the present study, values of the first correlation coefficient, *r*_1_, occurred in the interval [−0.5, 0.5] in steps of 0.01. For each value of *r*_1_, the second correlation value *r*_2_ occurred in the interval [(r_1_−0.5) (r_1_ + 0.5)] in steps of 0.01. For each cycle, *N* samples were simulated for each condition. As such, *N* will be the number of samples considered. *N* varied from 10 to 180, in steps of 2. As our focus was on the comparison of repeated measures (within-subject design without missing data), the number of samples for each condition was set to be equal; that is, *N* = n_1_ = n_2_. Correlation strengths and sample sizes were chosen in accordance with cognitive psychology- and neuroscience-like scenarios, but results can be extended to other research fields such as medicine and social psychology. Data were processed using MATLAB 9.2 (The Math Works Inc., Natick, MA). Statistics obtained for each method were averaged across values of *r*_1_ and zero-centered. Each plot shows the average statistics (e.g., *p*-value, effect size) with respect to increasing values of *r*_2_ and *N*, and across values of *r*_1_.

For LMEM and bootstrap models, sample data and covariate values were randomly generated and normally distributed, using the MatLab function *randn*. This led to the creation of two distributions for the dependent variable (DV), namely X_1_ and X_2_, with a mean μ_1_ = μ_2_ = 0 and a standard deviation σ_1_ = σ_2_ = 1. Independent variable (IV) and *N* covariate values were simulated for each condition and computed to yield the desired value of correlation with the DV. The covariates γ_1_ and γ_2_ were obtained as follows:


(1) $$\gamma_{i}=\sigma_{\gamma }\left(r_{i}*\chi_{i}+\sqrt{1-r_{i}^{2}}*\psi_{i}\right)+\mu_{\gamma }$$

where μ_γ_ and σ_γ_ are the desired mean and standard deviation of the covariate (with μ_γ_ = 0 and σ_γ_ = 1), respectively, while ψ is a pseudo-random set of *N* normally distributed values. This procedure was based on the Cholesky decomposition, which is commonly used in Monte-Carlo simulations of multiple correlated variables (Press et al., 1992). To note, since experimental psychology studies the relationship between variables in a sample, the method used is particularly appropriate for the case. In fact, it generates sample-level correlated data, and not population-level correlated data which would be an odd and non-realistic choice for simulations.

### Methods for Assessing Correlation Differences

#### Cohen’s q

Cohen proposed a simple method for interpreting the difference between two correlations (Cohen, 1988). Initially, to reduce skewness (asymmetry derived from the definition of *r*_*i*_ in the interval [−1, 1]), *r*-values were transformed to *z* values *via* the Fisher procedure:


(2) $$z_{i}=\left(0.5\right)\log\left(\frac{1+r_{i}}{1-r_{i}}\right)$$

Then, the absolute value of the difference between the two *z*-values was computed, such that *q* = |*z*_1_-*z*_2_|. The value *q* is the estimate of the effect size. The following intervals were proposed by Cohen to interpret these values: *q* < 0.1, *no* effect; 0.1 ≤ *q* < 0.3, *small* effect; 0.3 ≤ *q* < 0.5, *medium* effect; *q* ≥ 0.5, *large* effect. Since no *p*-value is associated with Cohen’s method, the only statistic reported is the effect size *q*.

#### Fisher’s Method

Fisher’s method (Fisher, 1921) is used to calculate the probability of two correlations being different, given the differences between *r*-values and the size of the two samples. The null hypothesis is that the correlation between X_1_ and γ_1_ will be the same as the correlation between X_2_ and γ_2_ for sample sizes n_1_ and n_2_. Correlation values *r*_1_ and *r*_2_ are converted to *z*-values as described in Equation 2. The test statistic *t* is then calculated:


(3) $$t=\frac{\left(z_{1}-z_{2}\right)}{\sqrt{1/\left({\text{n}}_{1}-3\right)+1/\left({\text{n}}_{2}-3\right)}}$$

Finally, using the cumulative distribution function of *t* in a standard distribution with mean μ_t_ = 0 and standard deviation σ_t_ = 1, the *p*-value is calculated to assess whether the null hypothesis can be trusted or not. Statistics reported for Fisher’s method are the *p*-value and *t*-statistic.

#### Linear Mixed-Effects Model

Linear mixed-effect models (LMEM) were applied in the form [DV ∼ IV*condition + (1| subject)] and thus included a fixed effect (the experimental condition), a continuous effect (the covariate IV), and a random intercept at the subject level (1| subject) to account for inter-individual variability. This type of model is applied frequently in psychology and neuroscience. At each cycle, a model was fitted using the MatLab function *fitlme* and the *p*-value and β statistics for the interaction between the experimental condition and the covariate were extracted.

Among applicable generalized linear models, the choice of the LMEM over, say, ANCOVA is due to the former’s increased flexibility and sensitivity (Schneider et al., 2015; Brysbaert and Stevens, 2018). Of note, as the aim of the present work is to investigate the power to predict an *effect*, corrections for multiple comparisons were unnecessary. Furthermore, since within-subject variability at the interaction level (subject: covariate) was not simulated, the introduction of random slopes was not necessary for the purposes of the study.

#### Bootstrap Method and Effect Size Estimation

The bootstrap method is a resampling technique often used to estimate confidence intervals and allows one to approximate the sampling distribution of a statistic (Efron and Tibshirani, 1986). In this study, we used a univariate, bias-corrected, accelerated bootstrap with replacement (Efron, 1987) to sample the correlation value *r*_*i*_. A sampling distribution was obtained by resampling the original data *k* times and obtaining *k* samples with sizes equal to the starting sample (*N*_*k*_ = *N*); that is, *k* is the number of bootstrap cycles. A similar sampling approach has been described for correlations (see Olkin and Finn, 1990, 1995); however, the method presented here implements a bias-corrected bootstrap procedure that can accommodate small sample sizes and outliers and includes an effect size estimation.

Each cycle was bootstrapped by estimating the correlation between the DV and covariate for each condition. Individually bootstrapping each correlation allows the estimation of the bootstrapped effect size for each condition, which may be useful for descriptive purposes. After *k* bootstrap cycles, two distributions of correlations were obtained for each condition and transformed into *z*-values. Each distribution possessed an associated mean and standard deviation (μ_*z*1_, σ_*z*1_; μ_*z*2_, σ_*z*2_). These distributions were then used for analyses. For each cycle (i.e., for each pair of r_2_ and *N*-values), the difference between the two *z*-distributions was represented as indicated in Equations 4 and 5, with the effect size estimated using Cohen’s *d* (Lipsey and Wilson, 2001; Ellis, 2010):


(4) $${\text{ES}}_{12}=d=\frac{\mu_{z1}-\mu_{z2}}{\sigma_{\text{pooled}}}$$

where


(5) $$\sigma_{\text{pooled}}=\sqrt{\frac{\left({\text{n}}_{1}-1\right)\sigma_{z1}^{2}+\left({\text{n}}_{2}-1\right)\sigma_{z2}^{2}}{{\text{n}}_{1}+{\text{n}}_{2}-2}}$$

A *Z*-test was performed comparing the distribution obtained by subtracting the two bootstrapped correlation distributions against a zero-centered distribution, to prevent biases caused by large sample sizes in estimating a *p*-value. Effect sizes (ES) can also be interpreted in terms of the percentage of non-overlap of the first group’s scores with those of the second group (Cohen, 1988). For example, ESs of 0.0, 0.8, and 1.7 indicate that the distribution of scores for the first group overlapped with the distribution of scores for the second group with 0, 47.45, and 75.4% of non-overlap, respectively.

#### Extension to Non-normal and Real Data

To generalize findings, the four methods were also applied on non-normally distributed data generated by taking the absolute values of normally distributed data, thus producing right-skewed (positively skewed) distributions. For consistency, the procedure adopted for the analyses of non-normally distributed data was the same as the one reported above.

Additionally, the four methods were also applied on behavioral data from the Human Connectome Project (HCP) database.^1^ More specifically, the same four methods described above were applied on behavioral data from 100 unrelated subjects which performed a language task (Binder et al., 2011). The task consists of two runs interleaving four blocks of a *story* condition and four blocks of a *math* condition. The story blocks present participants with brief auditory stories followed by a two-alternatives choice question in which participants are questioned about the topic of the story. The math task is adaptive with the aim of maintaining a similar level of difficulty and engagement across participants. The individual reaction time in the two conditions of the language task (within-subject levels: story, math) was used as the first variable. The second variable for correlation was taken from the battery of behavioral and individual difference measures and consisted of the age-adjusted score of *processing speed* as measured using the NIH Toolbox. This test measures the speed of cognitive processing of visually presented pairs of stimuli (Gershon et al., 2010). These measures were selected for the correlational analyses as they bring reliable and stable measurements on the population (Carlozzi et al., 2015; Wilson et al., 2016). Since the total sample size was *N* = 100, a Monte Carlo procedure was employed selecting randomly subsets of participants (from 10 to 100, with intervals of 10). Subgroups associated with each sample size ([10 20 30 40 50 60 70 80 90 100]) were analyzed 100 times, each time varying the randomly chosen subset of subjects.

## Results

**FIGURE 1:**
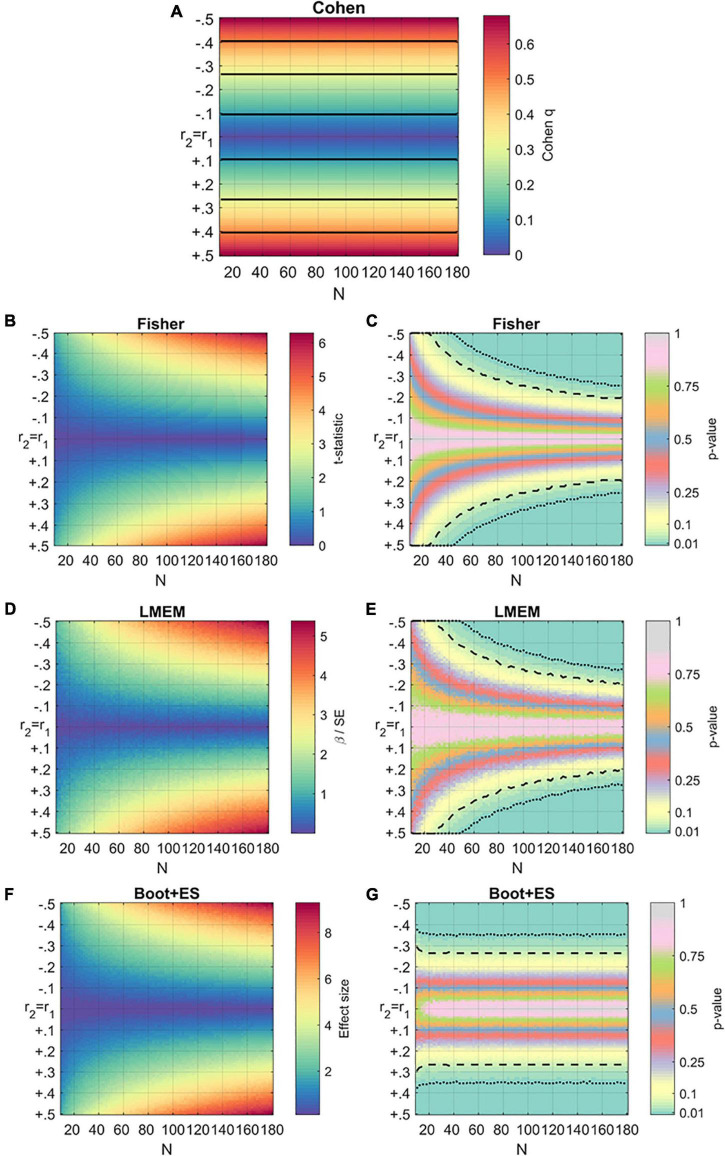
Results of the generalized simulations using the four methods. For all the subfigures, the horizontal axis represents the sample size, while the vertical axis represents values of r2 compared to r1. The third dimension (heat) represents the effect size or the *p*-value of each method **(A)** Results of the simulations using Cohen’s q. The effect sizes are averaged across values of r1. Black horizontal lines are used to separate the different levels of effect: small (blue), medium (cyan), large (green). **(B,C)** Results of the simulations using Fisher’s method. Statistics are averaged across values of r1. *T*-statistics are reported on the left panel, *p*-values on the right panel. Dashed and dotted lines represent thresholds of *p* < 0.05 and *p* < 0.01, respectively. **(D,E)** Results of the simulations using mixed-effect models (LMEM). Statistics are averaged across values of r1. β values for the interaction condition: covariate divided by their standard errors (SE) are reported on the left panel, corresponding *p*-values are reported on the right panel. **(F,G)** Results of the simulations using bootstrap (Boot + ES). Statistics are averaged across values of r1. Effect sizes (Cohens’ d) are reported on the left panel, corresponding *p*-values are reported on the right panel.

Results of the simulations are reported in Figure 1. In these plots, the x- and y-axes represent sample size and correlation difference, respectively, between *r*_1_ and *r*_2_. Values are averaged across different levels of *r*_1_ tested.

Figure 1A shows the results of Cohen’s *q* method, which returns an estimate of the effect (*q*) independent from the sample size *N*, reflecting only the difference between correlations. Empirically, small, medium, and large effects are defined based upon the magnitude of *q*. The interpretation of the result needs to be contextualized, however; a *large* difference between two correlations in a small sample (*N* < 10) is not systematically trustworthy. Conversely, a *small* effect might still be an important one (Rosnow and Rosenthal, 1989).

Figures 1B,C show *p*-values and *t*-statistics obtained using Fisher’s method. The test did not return significant values for relatively small sample sizes, even those with large differences between *r*1 and *r*2 (Δ*r* = 0.4). However, for larger samples (*N* = 40), this method failed to reject the null hypothesis as indicated by Δ*r* = 0.15. In the formula used for the *t*-statistic, sample size increases cause the test statistics to increase logarithmically, with the *p*-values showing a logarithmic decrease.

The mixed model analysis (LMEM) results are reported in Figures 1D,E. Like ANCOVA, mixed models are likely to fail to reject the null hypothesis even in the presence of a large difference between *r*1 and *r*2, as it interprets this difference as not being significant. The failure to reject the null hypothesis happens even with adequate sample sizes, such as an *N* of 40, by neuroscience standards. However, an advantage of LMEMs is that the means and standard deviations of the original data are retained. Results depend upon how the data points are distributed, and graphs tend to be more scattered than those described in previous paragraphs.

The *p*-value and effect size *d* obtained with bootstrapping simulations are reported in Figures 1F,G. Like in previous methods, *N* and *r*_2_ vary while *r*_1_ is fixed. The number (*k*) of random samplings was set to 200 (*k* = 200). Equivalent results were obtained for pilot simulations with *k* = 500 and *k* = 1,000; however, as these simulations included a reduced number of cycles for computational purposes, their results are not included here. As for Cohen’s method, the bootstrap approach provides an effect size estimation but not a *p*-value. Moreover, the procedure is sensitive to variability in the data, as indicated by the smoothness of the colors.

**FIGURE 2:**
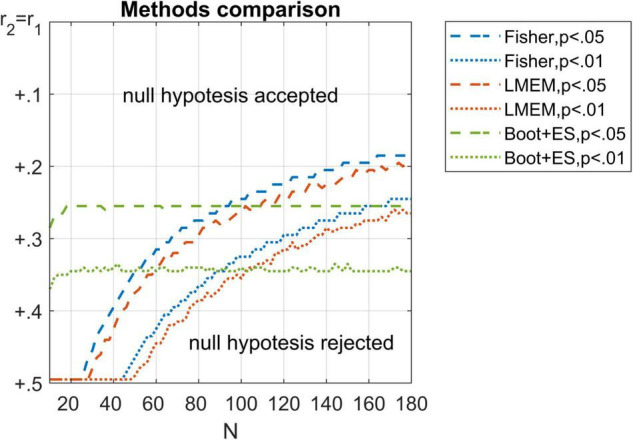
Comparison among Fisher (in blue), LMEM (in red), and bootstrap (in green) *p*-values. Thresholds of *p* = 0.05 (dashed lines) and *p* = 0.01 (dotted lines) are represented for each method. Only the lower half of the values are reported here for visual clarity.

A *post hoc* comparison among *p*-values gathered using Fisher, LMEM, and bootstrap methods is reported in Figure 2. The bootstrap method was relatively unaffected by the sample size, failing to reject more frequently the null hypothesis only with small samples (e.g., *N* < 20). Conversely, Fisher and LMEM failed to reject the null hypothesis even with significant differences between correlations, whereas the inverse bias was observed with large samples.

**FIGURE 3:**
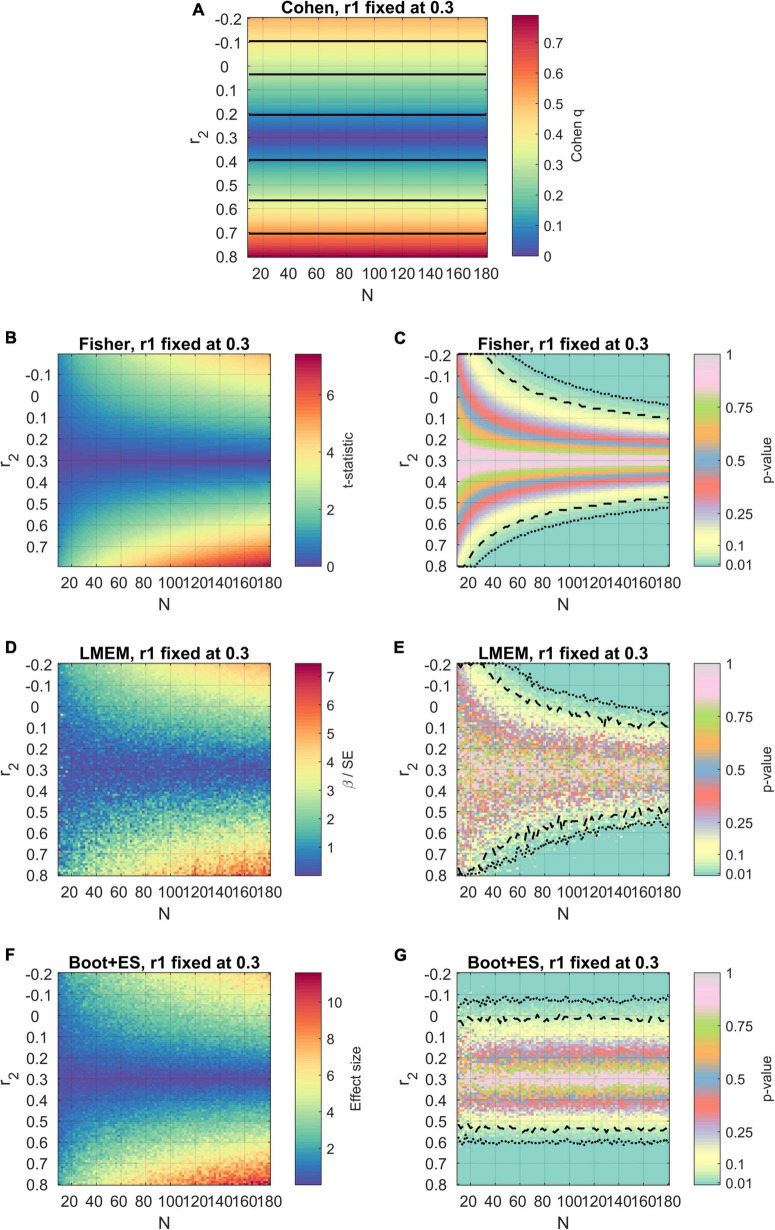
Results of the simulations using the four methods, with the first value of correlation fixed to *r*_1_ = 0.30. The axes, color scheme, and significance lines are the same as used in Figure 1. **(A)** Cohen’s method. **(B,C)** Fisher’s method. **(D,E)** LMEM. **(F,G)** Bootstrap followed by effect size estimation.

Since it may be pointed out that the results described until now may be based on averages across values of r1, an example with a specific value of r1 (r1 = 0.30) is illustrated in Figure 3. On the one hand, these results confirm the observations made until this point: Cohen’s method ignores sample size; Cohen’s and Fisher’s methods do not account for variability in the data structure; Fisher’s method and LMEM tend to fail to reject the null hypothesis even with significant differences in the correlational strength. However, the presence of overestimated effect sizes (see for example some points with *N* < 20 in Figure 3F for LMEM) shows that the variability in the data structure may endanger the estimation of fixed-effects statistics in mixed-models (Faraway, 2006), probably falling on Simpson’s paradoxes (Good and Mittal, 1987). The bootstrap procedure still accounted for variability in the data, but these paradoxical cases were not observed. This eventually happens because the bootstrap procedure limits the pitfalls of classical inferences methods (Killeen, 2005).

Results from the analysis of non-normally distributed data show overlapping results and are reported in Supplementary Material.

Finally, results from the analysis of real data confirm a different power of the four methods in detecting a reliable effect. Although the predictable effect of the processing speed on the readiness to respond to math problems, only the bootstrap procedure followed by the effect size estimation allowed to reject the null hypothesis, showing that the reaction time is negatively associated with processing speed only in the math condition of the language task (Table 1). Additionally, with this method it is observable a linear increase of the effect size with the sample size, which is a desirable propriety of a statistical method for studying interindividual variability in psychometric and neuroscientific experiments.

**TABLE 1:** Results from the correlational analyses of the real dataset including a task-performance variable (reaction time during language-story and language-math task conditions) and a cognitive “baseline” variable (processing speed as assessed using the NIH Toolbox).

	Cohen	Fisher		LMEM		Boot + ES	
N	*q*	*t*-stat	*p*	β	*p*	*d*	*p*
10	0.33	0.42	0.59	4.63	0.47	0.97	0.065
20	0.29	0.80	0.48	6.54	0.39	1.37	0.103
30	0.23	0.83	0.46	5.52	0.36	1.38	0.094
40	0.23	0.99	0.38	5.93	0.29	1.59	0.052
50	0.23	1.11	0.31	5.81	0.22	**1.81**	**0.024**
60	0.24	1.26	0.25	5.96	0.16	**2.00**	**0.009**
70	0.23	1.31	0.22	5.80	0.14	**2.08**	**0.009**
80	0.24	1.46	0.16	6.06	0.09	**2.31**	**0.002**
90	0.23	1.48	0.15	5.79	0.08	**2.34**	**0.002**
100	0.22	1.56	0.12	5.81	0.06	**2.45**	**<0.001**

*Bold values indicate significant effects (p < 0.05).*

## Discussion

The present study compared vintage and modern statistical methods used to evaluate differences across correlations in the fields of psychology, medicine, and related disciplines.

A direct comparison between correlation coefficients as provided by Cohen’s method can be helpful, given that the calculation only requires the two correlation values (Cohen, 1988). However, Cohen’s method estimates the effect magnitude irrespectively of sample size. Conversely, the effect estimated by Fisher’s method increases with increasing sample size. Although these two methods can be useful when the original data structure is unavailable, neither Fisher’s nor Cohen’s method consider potential variability in the data. The effects estimated with these methods should be carefully contextualized and interpreted.

It has been suggested that LMEMs may generate *p*-values that are too small, possibly overestimating the importance of a given effect (Faraway, 2006). The primary purpose of the LMEM is to use data from a continuous variable to estimate differences between levels of a fixed factor, including repeated-measures or longitudinal scenarios (Robinson, 1991). Given the results presented here, LMEM (and other generalized linear models, like the ANCOVA) may be useful for explorative purposes. Still, they may not be the best choice if the aim is to test the difference between the two correlations. The presence of overestimated effect sizes for LMEM suggested possible miscalculation on fixed-effects coefficients in mixed-models (Good and Mittal, 1987; Faraway, 2006).

The bootstrap procedure followed by a test to determine effect size accurately estimated the difference between the strength of the two correlations. Key features of this method are that it considers the sample size and the variability of the initial data, returns a descriptive measure of the difference between the two correlations, and provides a *p*-value not biased by small or large sample sizes and not affected by the pitfalls of classical inferences methods (Killeen, 2005).

Although Fisher, LMEM, and bootstrap methods returned a *p*-value, which is traditionally used to assess the presence or absence of an effect, the debate about how and even if these statistics should be used persists (Goodman, 2019). Due to logistical constraints, certain seminal cognitive neuroimaging studies dealt with relatively small samples (Lindquist et al., 2012; Friston, 2013). Such sample sizes may not be enough to detect significant differences using linear regression (LMEM). Recently, *p*-values have been shown to be misleading measures of the strength of the evidence against the null hypothesis (Berger and Sellke, 1987; Hupé, 2015); also, they do not directly provide an index of effect magnitude (Sullivan and Feinn, 2012). The bootstrap approach provided a descriptive effect size close to the effect estimated by mixed models. The efficiency of the bootstrap approach in this study is in line with the trend toward *p*-independent assays in psychology (Ellis, 2010; Kelley and Preacher, 2012; Tabachnick and Fidell, 2012; Nichols et al., 2017). Furthermore, implementing a *z*-test provided an unbiased, universally used null-hypothesis statistic that may still be useful for *p*-generation researchers (Greenland, 2019; Ioannidis, 2019). Bootstrapping correlations have been discussed extensively in the literature, and researchers have noted that monotonic, transformation invariant procedures like the bootstrap are ill-suited to estimating confidence intervals or testing a null hypothesis (Lunneborg, 1985; Efron and Tibshirani, 1986; Rasmussen, 1987; Efron, 1988; Strube, 1988; Olkin and Finn, 1995). However, the procedure presented here and compared with other methods represents a slightly new approach that combines bootstrap with effect size estimation and null-hypothesis testing.

Importantly, the application to real data confirmed the usefulness of the bootstrap approach. In fact, the Monte-Carlo procedure revealed that the bootstrap followed by the effect size estimation was the only method able to reject the null hypothesis across many sample sizes. Through this method, that the cognitive trait of processing speed significantly predicted reaction time during the “math” condition but not during the “story” condition of a language task. Furthermore, only with the bootstrap method followed by the effect size estimation the strength of the effect regularly increased with increasing sample sizes, reflecting the accumulation of evidence with increasing levels of information.

## Conclusion

The present study evaluated the efficacy of four different methods for investigating differences in correlations across experimental conditions. Bootstrapping followed by effect size estimation was the most successful, providing a statistic that accounted for both inter-individual and sample size variability in comparing correlation coefficients between experimental conditions. This method is easily implementable in MatLab through the bootes function made available online by the author at MathWorks.

Although these findings have implications for researchers interested in comparing the magnitude of correlations between different experimental conditions, this study has a significant limitation that must be acknowledged. In fact, the bootstrap procedure presented here works well for within-subject analyses and may be applied without complications to between-subjects paradigms but is not yet applicable to mixed experimental designs which include both between- and within-subject factors. Future studies should evaluate these and other methods in such alternative situations to uncover other easily implemented, bias-free tools for researchers in psychology, neuroscience, and medicine.

## Data Availability Statement

The original contributions presented in the study are included in the article/Supplementary Material, further inquiries can be directed to the corresponding author/s.

## Author Contributions

SD ideated the study, performed simulations, wrote the manuscript, produced the figures, and wrote the program “bootes.”

## Conflict of Interest

The author declares that the research was conducted in the absence of any commercial or financial relationships that could be construed as a potential conflict of interest.

## Publisher’s Note

All claims expressed in this article are solely those of the authors and do not necessarily represent those of their affiliated organizations, or those of the publisher, the editors and the reviewers. Any product that may be evaluated in this article, or claim that may be made by its manufacturer, is not guaranteed or endorsed by the publisher.

## Footnotes

1
balsa.wustl.edu
