# Supplementary material for: Testing the Magnitude of Correlations Across Experimental Conditions
Source: Front Psychol. 2022 May 26;13:860213. doi: 10.3389/fpsyg.2022.860213 (PMC9177411; doi:10.3389/fpsyg.2022.860213)
Supplement: Supplementary file 1 [file Data_Sheet_1.PDF]

SUPPLEMENTARY MATERIAL

Testing the magnitude of correlations across experimental conditions

Simone Di Plinio[1]*

1 Department of Neuroscience Imaging and Clinical Sciences, "G. D'Annunzio" University of Chieti-Pescara, Chieti 66100, Italy

* Corresponding author: Simone Di Plinio; ORCID: 0000-0002-2551-0985, Department of Neuroscience Imaging and Clinical Sciences, "G. D'Annunzio" University of Chieti-Pescara, Chieti 66100, Italy. simonediplinio@yahoo.it

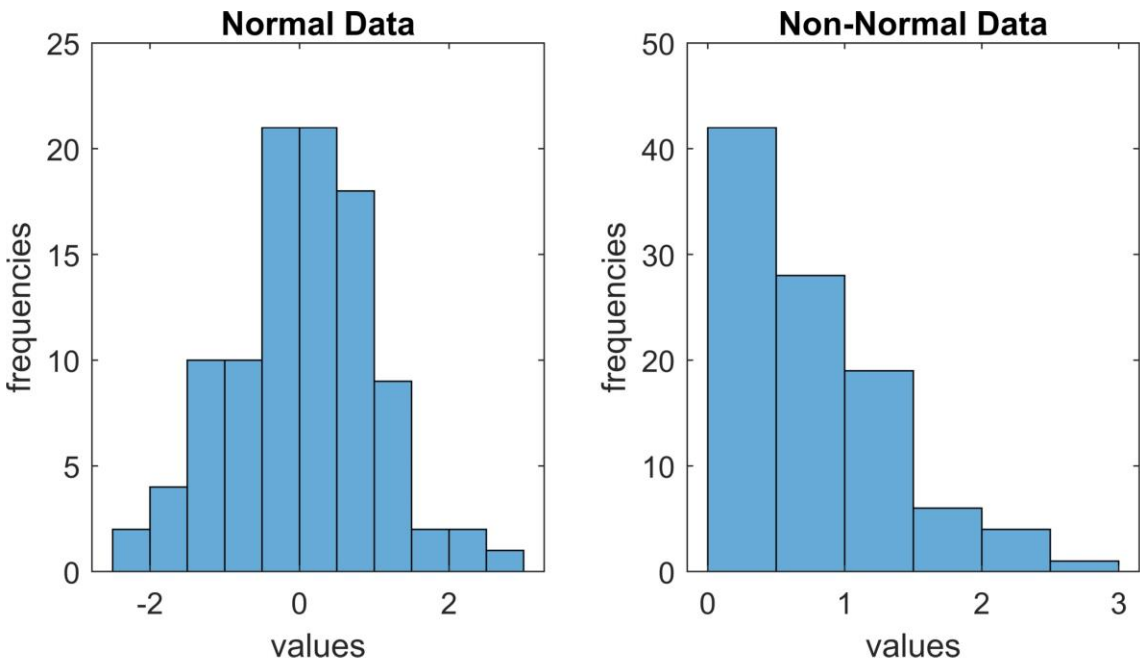

Figure S1. Visualization of an example of normally distributed data (left) and non-normally distributed, right-skewed data (right) obtained by taking the absolute values from the original normal distribution.

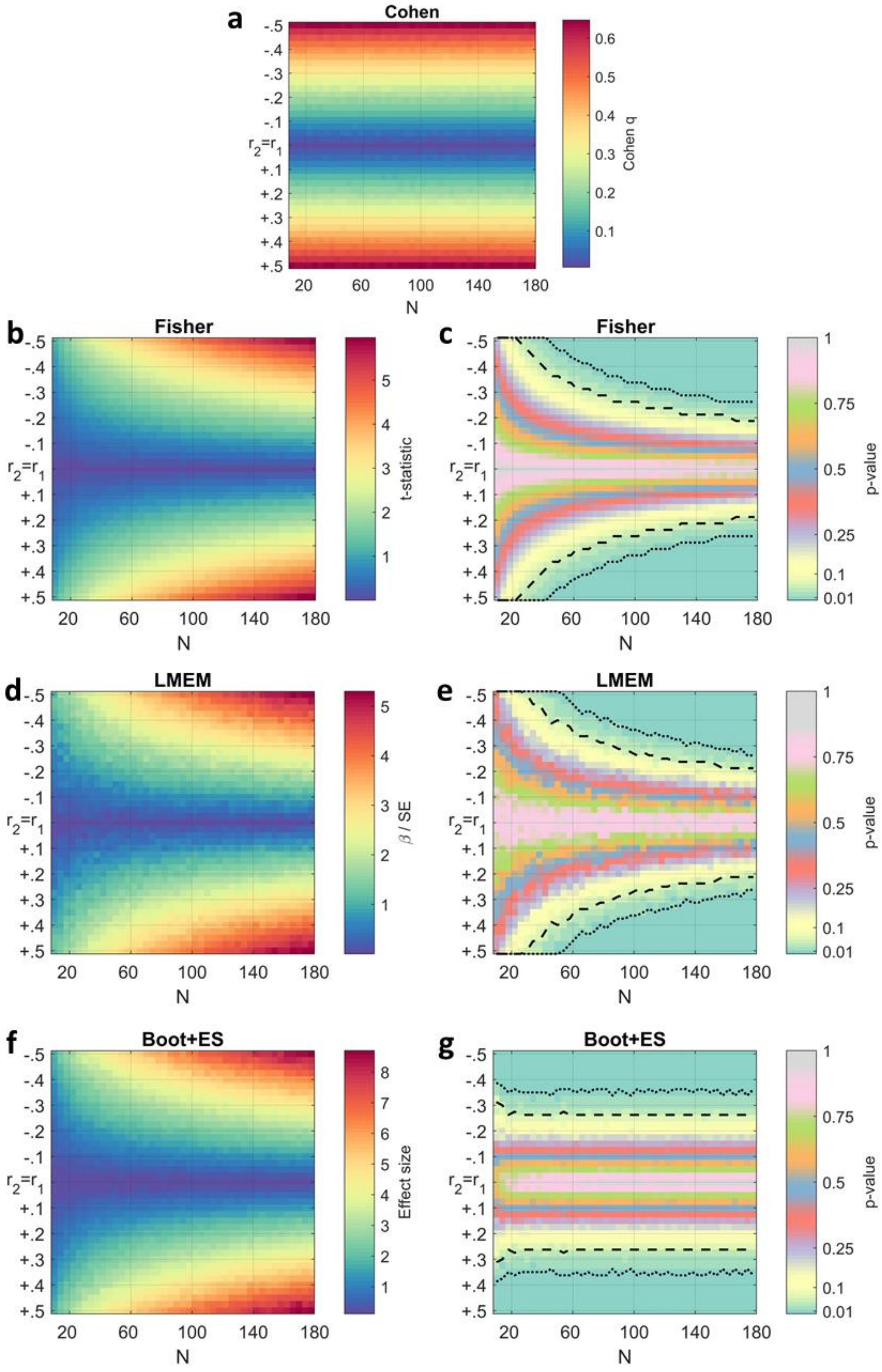

Figure S2. Results of the simulations using the four methods on non-normally distributed data. For all the subfigures, the horizontal axis represents the sample size, while the vertical axis represents values of r2 compared to r1. The third dimension

(heat) represents the effect size or the p-value of each method (a) Results of the simulations using Cohen's q. The effect sizes are averaged across values of r1. Black horizontal lines are used to separate the different levels of effect: small (blue), medium (cyan), large (green). (b-c) Results of the simulations using Fisher's method. Statistics are averaged across values of r1. T-statistics are reported on the left panel, p-values on the right panel. Dashed and dotted lines represent thresholds of $p<0.05$ and $p<0.01$, respectively. (d-e) Results of the simulations using mixed-effect models (LMEM). Statistics are averaged across values of r1. β values for the interaction condition : covariate divided by their standard errors (SE) are reported on the left panel, corresponding p-values are reported on the right panel. (f-g) Results of the simulations using bootstrap (Boot+ES). Statistics are averaged across values of r1. Effect sizes (Cohens' d) are reported on the left panel, corresponding p-values are reported on the right panel.

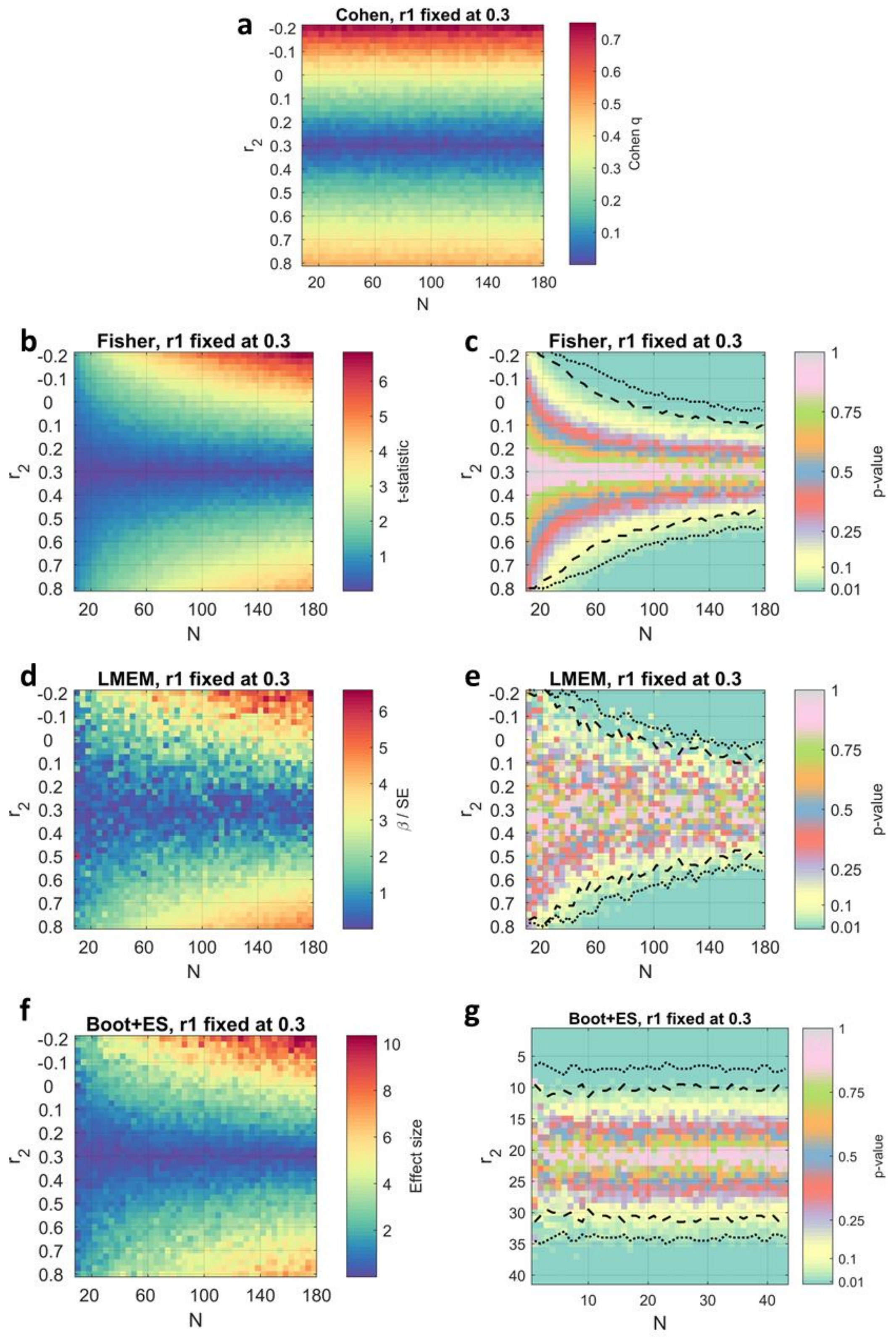

Figure S3. Results of the simulations using the four methods on non-normally distributed data, with the first value of correlation fixed to r_1=0.30. **(a)** Cohen's method. **(b-c)** Fisher's method. **(d-e)** LMEM. **(f-g)** Bootstrap followed by effect size estimation.
